# EIF3C-enhanced exosome secretion promotes angiogenesis and tumorigenesis of human hepatocellular carcinoma

**DOI:** 10.18632/oncotarget.24149

**Published:** 2018-01-11

**Authors:** Hsin-Yi Lee, Chi-Kuan Chen, Chun-Ming Ho, Szu-Shuo Lee, Chieh-Yu Chang, Kuan-Ju Chen, Yuh-Shan Jou

**Affiliations:** ^1^ Graduate Institute of Life Sciences, National Defense Medical Center, Taipei, Taiwan; ^2^ Institute of Biomedical Sciences, Academia Sinica, Taipei, Taiwan; ^3^ Department of laboratory medicine, MacKay Memorial Hospital, Taipei, Taiwan; ^4^ School of medicine, MacKay Medical College, Taipei, Taiwan; ^5^ Institute of Bioinformatics and Systems Biology, National Chiao Tung University, Hsinchu, Taiwan; ^6^ Bioinformatics Program, Taiwan International Graduate Program, Academia Sinica, Taipei, Taiwan; ^7^ Program in Molecular Medicine, National Yang-Ming University and Academia Sinica, Taipei, Taiwan; ^8^ Taiwan International Graduate Program in Molecular Medicine, National Yang-Ming University and Academia Sinica, Taipei, Taiwan; ^9^ Graduate Institute of Microbiology, College of Medicine, National Taiwan University, Taipei, Taiwan

**Keywords:** Hepatocellular carcinoma, EIF3C, exosome, angiogenesis, S100A11

## Abstract

Targeting tumor angiogenesis is a common strategy against human hepatocellular carcinoma (HCC). However, identification of molecular targets as biomarker for elevating therapeutic efficacy is critical to prolong HCC patient survival. Here, we showed that EIF3C (eukaryotic translation initiation factor 3 subunit C) is upregulated during HCC tumor progression and associated with poor patient survival. Expression of EIF3C did not alter proliferation and expression of other tumor progressive genes such as HIF1A, TGFβ1 and VEGF, but reduced cell migration in HCC cells. Nevertheless, expression of EIF3C in HCC cells significantly increase secretion of extracellular exosomes confirmed by increased exosomes labelling by PKH26 fluorescent dye, vesicles in exosome size detected by electronic microscopy and nanoparticle tracking analysis, and expression of divergent exosome markers. The EIF3C-increased exosomes were oncogenic to potentiate tumor angiogenesis via tube formation of HUVEC cells and growth of vessels by plugs assays on nude mice. Subcutaneous inoculation of EIF3C-exosomes mixed with Huh7 HCC cells not only promoted growth of vessels but also increased expression of EIF3C in tumors. Conversely, treatment of exosome inhibitor GW4869 reversed aforementioned oncogenic assays. We identified EIF3C activated expression of S100A11 involved in EIF3C-exosome increased tube formation in angiogenesis. Simultaneous high expression of EIF3C and S100A11 in human HCC tumors for RNA level in TCGA and protein level by IHC are associated with poor survival of HCC patients. Collectively, our results demonstrated that EIF3C overexpression is a potential target of angiogenesis for treatment with exosome inhibitor or S100A11 reduction to suppress HCC angiogenesis and tumorigenesis.

## INTRODUCTION

Hepatocellular carcinoma (HCC) is the third leading cause of cancer death in the world with more than 788,000 death per year based on data presented in the World Health Organization (WHO). HCC is the most common liver malignancy with risk factors to the liver such as chronic viral hepatitis of B or C, exposure of alcohol abuse and aflatoxin toxins, and aberrant metabolic stress such as nonalcoholic steatohepatitis (NASH) and obesity [1]. Less than 50% of HCC patients were diagnosed at early stage and majority of them were not qualified for curable surgical resection owing to late stage of tumor [2]. The prognosis for untreated HCC patients has a poor average survival rate 6∼20 months and more than 50% of treated patients developed recurrence and metastasis within 5 years of therapy [3]. A multiple kinase inhibitor Sorafenib was firstly approved for systemic therapy of advanced HCC in 2007 with an average response rate of 2∼3% and extending patient survival for a few months [4]. Another multiple kinase inhibitor regorafenib showed 10∼20% response rate as second-line therapy for advanced HCC was approved by FDA in 2017 [5]. Both multiple kinase inhibitors showed anti-angiogenesis activity by targeting angiogenic and oncogenic receptor tyrosine kinases with modest improvement of HCC patient survival [6]. Moreover, with limited success on HCC cancer genome sequencing for common mutations as therapeutic targets for drug development, it is critical to develop other innovative strategies and novel targets and drugs to prolong survival of HCC patients [7].

Several lines of evidence already supported that aberrant translational machinery such as modulation of ribosome biogenesis, Akt1/mTOR signaling and translational initiation play critical roles in tumor progression [8, 9]. Translational initiation is the rate-limiting step of protein synthesis that could alter the overall gene expression or selectively enhance translation of oncogeneic mRNAs to impact cancer development and progression [10, 11]. EIF4E, the best-studied eukaryotic initiation factor (eIF), is frequently overexpressed in cancers and activates Akt1/mTOR signaling pathway to phosphorylate 4EBP1 (eukaryotic translation initiation factor 4E binding protein 1) for releasing EIF4E to bind to 5′ cap of mRNA to enable translation of oncogenic genes [12, 13]. EIF3 translation initiation complex is the largest eIF protein complex composed of 13 subunits from eIF3a to eIF3m and orchestrates formation and stability of 43S preinitiation complexes (PICs) for translational initiation [14]. Aberrant expression of eIF3 subunits were reported in many malignant tumors and play important roles during tumor progression [15, 16]. EIF3C also known as EIF3S8 or eIF3-p110 were found to be upregulated in neurofibromatosis type 2 (NF2), colon cancer, glioma, HCC and breast cancer. Silencing of EIF3C via knockdown or interacting with schwannomin could induce cell apoptosis and suppress cell proliferation and tumor growth [17–23]. Nevertheless, the underlining molecular mechanisms for upregulated EIF3C to mediate tumor progression and serve as therapeutic target to improve patient survival remain unclear.

Extracellular vesicles were released as heterogeneous plasma membrane vesicles from majority of cell types into body fluids under normal or disease conditions for intercellular communication [24]. Based on their size and biogenesis, extracellular vesicles in diameter could be mainly divided into exosomes (30∼150 nm), microvesicles (or microparticles) (100 nm∼1 µm) and apoptotic bodies (>1 µm) as cargos for transmitting proteins, lipids, coding RNAs, noncoding RNAs such as microRNAs or even DNA to target cells for inducing various signaling cascades [25]. Numerous studies have shown that cancer cells utilized extracellular vesicles to communicate with stroma cells in the tumor microenvironment similar to cytokines and growth factors VEGF, FGF, TGFβ and Wnt to sustain favorite microenvironment for tumor progression, angiogenesis and metastasis [26, 27]. As part of routing clinical liquid biopsy, extracellular vesicles especially nano-sized exosomes could carry various cargos such as proteins, lipids, mRNAs, and miRNAs, and be easily uptook by recipient cells are emerging for developing tools for diagnostic and therapeutic interventions [28, 29].

In this study, we explored the roles of EIF3C upregulation in the tumor progression of HCC. Rather than direct stimulation of tumorigenic features such as cell proliferation and cell migration, we found an interesting mechanism that expression of EIF3C in HCC cells increase secretion of exosomes to promote angiogenesis and tumorigenesis of HCC with cross-validations of markers in human HCC tumor samples.

## RESULTS

### EIF3C is overexpressed and associated with poor patient survival in multiple HCC cohorts

**Figure 1:**
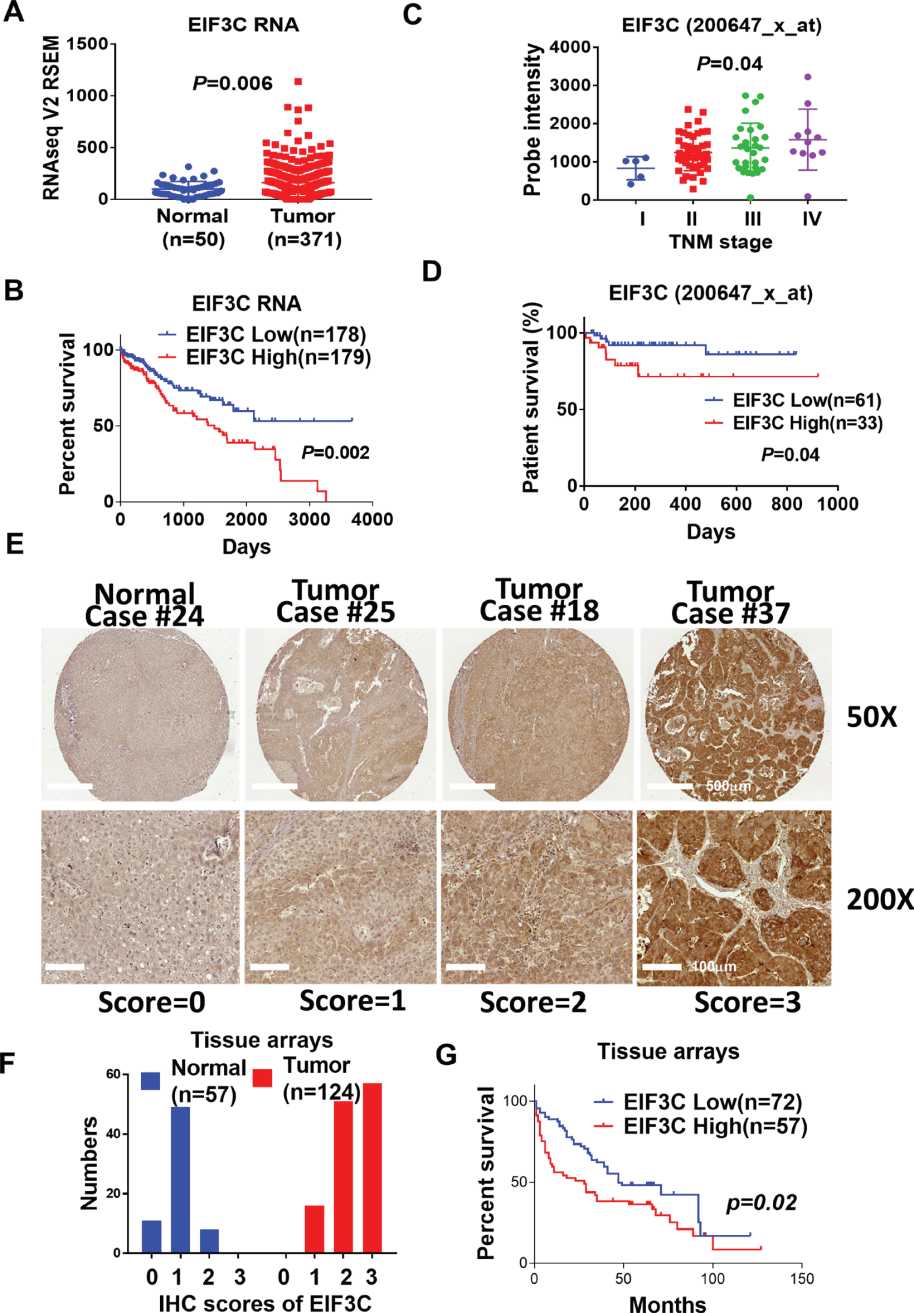
EIF3C is overexpressed and associated with poor patient survival in multiple HCC cohorts (**A**) EIF3C RNA expression is higher in tumor than that of normal tissues in TCGA dataset (Unpaired *t* test). (**B**) Higher expression of EIF3C is associated with poor survival than lower expression of EIF3C in TCGA HCC dataset. (Log-rank (Mantel-Cox) test). (**C**) EIF3C expression is upregulated during HCC tumor progression (Probe:200647_x_at, iCOD dataset) (Bartlettʼs test). (**D**) EIF3C overexpression is associated with poor patient survival (Probe:200647_x_at, iCOD dataset). (Log-rank (Mantel-Cox) test). (**E**) Representative EIF3C protein expression level in normal and tumor sample in tissue arrays for scoring from 0 to 3 by immunohistochemistry (IHC) assays. (**F**) EIF3C protein expression distribution in normal and tumor tissues after IHC scoring from 0 to 3. (**G**) Higher protein expression of EIF3C (IHC score: 3, *n* = 57) is associated with poor survival in compared to lower expression of EIF3C (IHC score: 0, 1 and 2, *n* = 72) in HCC tumor tissues in tumor tissue arrays. (Log-rank Mantel-Cox test).

We found that EIF3C is upregulated in HCC tumor samples in comparison with normal tissues in TCGA HCC dataset (Figure 1A). Higher expression of EIF3C in tumor samples is associated with poor patient survival in compared to that of lower expression HCC patients (Figure 1B). We validated EIF3C RNA expression in another HCC transcriptome dataset and found that EIF3C gradually increased expression to advanced stages and associated with poor patient survival of HCC patients [30] (Figure 1C and 1D). Consistently, we also detected higher expression of EIF3C protein level at advanced stages of HCC tissues and in association with poor patient survival by using immunohistochemistry (IHC) assays (Figure 1E–1G).

The increasing expression of EIF3C in HCC tissues prompted us to examine its oncogenic properties in HCC cells. We were disappointed that there is no alterations in cell proliferation and expression of tumor progression-related genes including HIF1A, TGFβ1 and VEGF at RNA levels but decrease of trans-well cell migration assays in HCC cells PLC5, SNU449 and Huh7 (Supplementary Figure 1A–1C).

### Overexpressed-EIF3C in HCC cells increased release of exosomes and enhanced angiogenesis *in vitro* and *in vivo*

**Figure 2:**
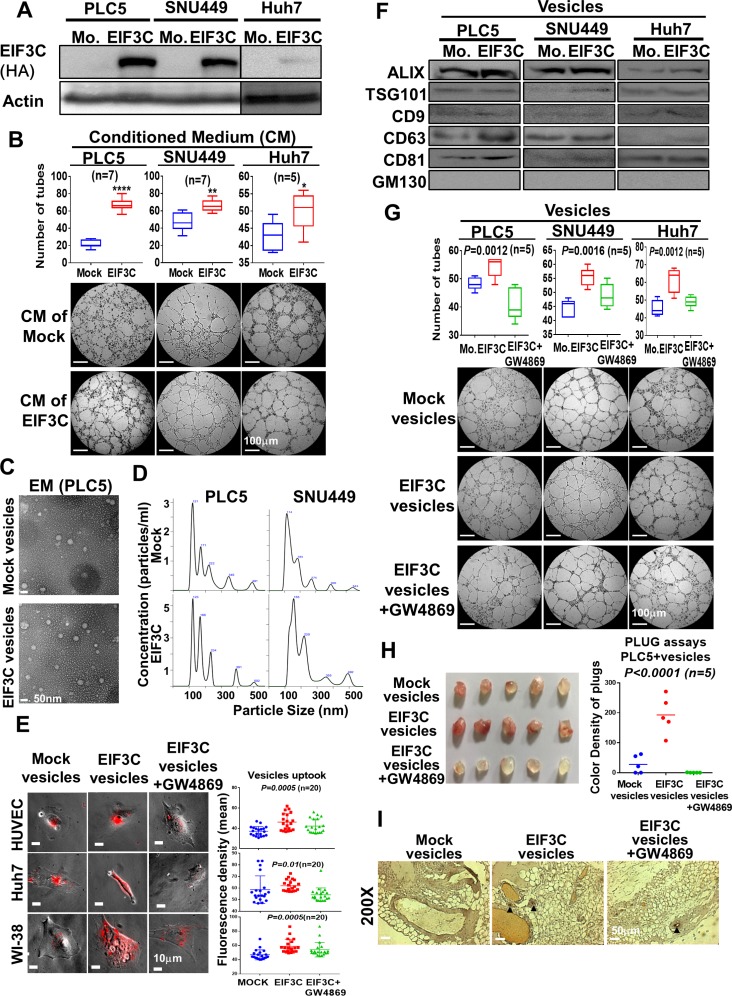
Overexpression of EIF3C in HCC cells increased secretion of exosomes to promote HCC angiogenesis *in vitro* and *in vivo* (**A**) Western analysis of EIF3C expression in HCC cell lines PLC5, SNU449 and Huh7. (**B**) Conditioned mediums collected from EIF3C expressed HCC cells enhanced angiogenic tubes formation of HUVEC cells. (**C**) Observation of vesicles in EIF3C-expressed PLC5 in compared to that of mock PLC5 by electron microscopy (EM). (**D**) Typical images produced by the NanoSight technique. Particle size distribution and calculated concentration in the vesicles of mock and overexpressed EIF3C samples. (**E**) HUVEC (endothelial cells), Huh7 (epithelial cells) and WI-38 (fibroblasts) cells up took more PKH26 labelled vesicles from EIF3C-released than that of mock cells. (ANOVA summary). (**F**) Western blotting analysis of exosome markers ALIX, TSG101, CD9, CD63 and CD81 in purified exosomes of HCC cells with and without EIF3C expression. GM130 served as negative control. We detected GM130 in total cell lysates for positive antibody control. (**G**) Tubes formation of HUVEC angiogenesis assays by treatments of vesicles of mock, EIF3C expression and EIF3C expression co-treated with exosome inhibitor GW4869. (**H**) Plugs assays for *in vivo* angiogenesis with PLC5 vesicles of mock, EIF3C expression and EIF3 expression co-treated with GW4869. (**I**) CD31 expression by IHC assays of plugs generated from PLC5 vesicles of mock, EIF3C expression and EIF3C expression co-treated with GW4869.

To investigate the roles of overexpressed EIF3C in HCC progression, we performed proteomic study of EIF3C protein complex with immunoprecipitation by using mass spectrometry. Interestingly, we found that EIF3C pulled down 1,738 proteins that participated in functions such as intracellular trafficking and secretion in the clusters of orthologous groups (COGs) and extracellular exosome in cellular component of gene ontology by DAVID Bioinformatics analysis (Supplementary Figure 2A and Supplementary Table 1). We hypothesized that EIF3C might enhance secretion of exosomes to promote tumor angiogenesis. We found that incubation of conditioned mediums collected from EIF3C-overexpressed HCC cells PLC5, SNU449 and Huh7 (Figure 2A) with HUVEC cells could enhance the tube formation of HUVEC in compared with that of mock control for *in vitro* angiogenesis assays (Figure 2B). We speculated that the conditioned mediums especially collected from EIF3C-expressed HCC cells might increase the release of vesicles to promote tube formation of HUVEC cells. Indeed, more vesicles ranged in exosome size were observed in EIF3C overexpressed PLC5 than that of mock control detected by electron microscopy (EM) (Figure 2C) [31] and nanoparticle tracking analysis (NTA) (Figure 2D) [32, 33]. Consistently, when incubated HUVEC cells with PKH26-labelled vesicles, more PKH26-labelled vesicles derived from EIF3C expressed PLC5 were uptook by HUVEC cells than that of mock control (Figure 2E). Moreover, PKH26 labelled vesicles of EIF3C-expressed PLC5 were uptook more than that of mock control in terms of fluorescent labelling intensity in various HCC epithelial cells (Huh7, SNU449 and PLC5) and fibroblasts (WI-38 and NIH3T3) (Figure 2E and Supplementary Figure 2B–2C) [34]. Together, our results demonstrated that EIF3C-overexpressed HCC cells could increase the release of extracellular exosomes and uptake by divergent cell types.

To confirm EIF3C expression increased release of exosomes in HCC cells, we detected protein expression of exosome biomarkers in exosomes including ALIX, TSG101, CD9, CD63 and CD81 as positive as well as GM130 as negative controls by Western blotting analysis [35–37]. To evaluate angiogenic functions of EIF3C-enhanced exosomes, we performed tubes formation of HUVEC cells *in vitro* and plugs assays *in vivo* for angiogenesis assays with PLC5 vesicles under treatments of exosome generation inhibitor GW4869 [38]. Our results demonstrated that EIF3C slightly increased expression of divergent exosome biomarkers in vesicles of HCC cells (Figure 2F), increased RNA concentration ratios in exosomes (Supplementary Figure 2E), enhanced tubes formation of HUVEC cells (Figure 2G) and promoted the formation of new blood vessels in the transplanted matrix gel plugs in nude mice (Figure 2H and 2I). In contrast, treatment of exosome generation inhibitor GW4869 in EIF3C expressed HCC vesicles diminished aforementioned exosome uptook, biomarker expression, and angiogenesis *in vitro* and *in vivo* (Figure 2E, 2G–2I and Supplementary Figure 2B–2D). We further performed exosome proteomic analysis for links of EIF3C-mediated angiogenic functions by using mass spectrometry and gene ontology analysis by DAVID Bioinformatics. Consistently, our results showed that the 204 EIF3C specific exosome containing proteins might participate in extracellular exosome in cellular component of gene ontology analysis and in VEGF, hypoxia and angiogenesis of BIOCARTA pathway (Supplementary Figure 2F and Supplementary Table 2). Together, our results demonstrated that EIF3C- overexpressed HCC cells could increase the release of extracellular exosomes to promote *in vitro* angiogenesis by tube formation assays.

### Inoculation of EIF3C-increased PLC5 exosomes with Huh7 enhanced HCC angiogenesis and tumorigenesis

**Figure 3:**
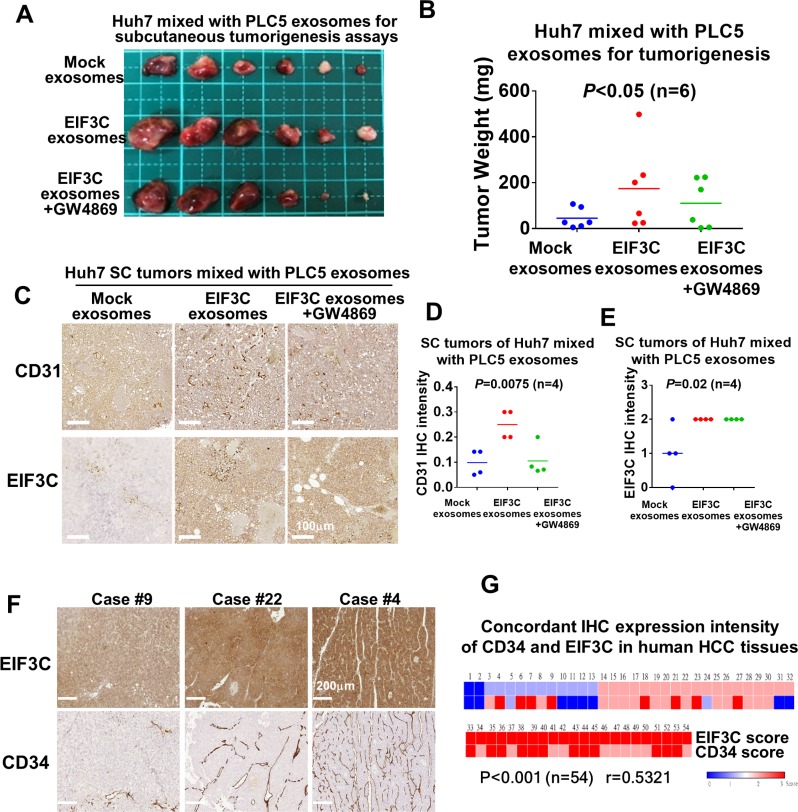
Various exosomes isolated from PLC5 mixed with Huh7 cells enhanced HCC angiogenesis and tumorigenesis (**A**) Subcutaneous tumorigenesis assays of Huh7 cells mixed with exosomes isolated from mock, EIF3C expression and EIF3C expression co-treated with GW4869. (**B**) Tumor weight and summary of subcutaneous tumorigenesis assays of Huh7 cells mixed with PLC5 exosomes isolated from mock, EIF3C expression and EIF3C expression co-treated with GW4869 (ANOVA summary). (**C**) Representative IHC staining of CD31 and EIF3C expression in PLC5 exosomes-enhanced subcutaneous Huh7 tumors with and without EIF3C expression and co-treatment of GW4869. (**D**) PLC5/EI3C exosomes enhanced CD31 expression is suppressed in compared to GW4869- treated PLC5/EIF3C exosomes-mediated Huh7 SC tumors (ANOVA summary). (**E**) Expression of EIF3C in PLC5/EIF3C-mediated Huh7 subcutaneous tumors showed no difference in compared to with and without treatments of GW4869 (ANOVA summary). (**F**) Representative IHC staining of EIF3C and angiogenic marker CD34 in human HCC tumors. (**G**) Heat map of concordant expression of CD34 angiogenic marker with EIF3C by IHC assays of HCC patients.

In addition to enhance HCC angiogenesis, we examined the potential of EIF3C-increased exosomes participated in HCC tumorigenesis. Interestingly, we found that subcutaneous inoculation of mixture of EIF3C-enhanced PLC5 exosomes with Huh7 into nude mice promoted tumor growth (Figure 3A and 3B). The increased tumor masses was suppressed by treatment of exosome inhibitor GW4869 on the EIF3C enhanced exosomes in the inoculated mixture with Huh7 cells in the subcutaneous tumorigenesis assays. Moreover, we also found that the increased subcutaneous tumor growth of the mixture of EIF3C-enhanced exosomes with Huh7 also potentiated expression of EIF3C and CD31 endothelial marker by IHC assays (Figures 3C-E). Treatments with exosome inhibitor GW4869 suppressed expression of CD31 endothelial marker but not EIF3C expression in the subcutaneous tumors.

To validate the expression of EIF3C in promoting human HCC tumorigenesis and angiogenesis, we performed IHC assays for concordant expression of EIF3C and vessel biomarker CD34 in human HCC tumors. We found that upregulated-EIF3C expression is concordantly increased expression of CD34 endothelial marker in human HCC tumors (*P <* 0.001 and *r =* 0.5321) (Figure 3F and 3G). Our results suggested that the increasing expression of EIF3C in HCC cells could promote HCC angiogenesis and tumorigenesis via increasing secretion of exosomes.

### S100A11 involved in EIF3C-enhanced angiogenesis and tumorigenesis of HCC

**Figure 4:**
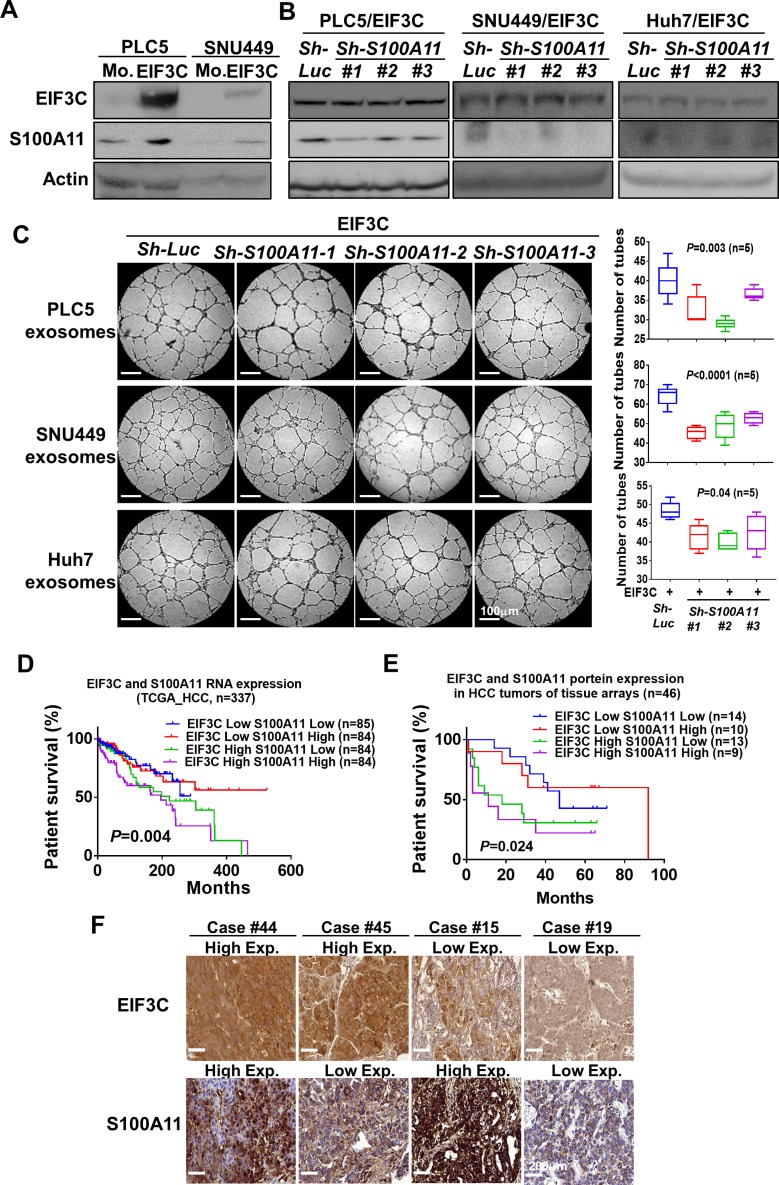
S100A11 involved in EIF3C exosome-enhanced angiogenesis and tumorigenesis in HCC (**A**) Expression of EIF3C increased expression of S100A11 in HCC cells by Western Blotting analysis. (**B**) Knockdown S100A11 in EIF3C expressed HCC cells in compared to control knockdown did not alter expression of EIF3C. (**C**) Exosomes collected from EIF3C expressed and S100A11 knockdown HCC cells reduced EIF3C vesicle enhanced HUVEC angiogenesis. (**D**) Simultaneous RNA expression of EIF3C and S100A11 in HCC of TCGA dataset associated with poor patient survival. (**E**) Simultaneous protein expression of EIF3C and S100A11 by IHC assays in HCC patients associated with poor patient survival. (**F**) Representative IHC staining of EIF3C and S100A11 protein expression divided by high and low expression in HCC tumors.

We found that two members of S100 family proteins S100A11 and S100P were identified in EIF3C exosome proteomic data (Supplementary Table 2). Since members of S100 family proteins are known to play critical roles in cancer progression and angiogenesis [39–41] and since recent quantitative proteomics of exosomes identified S100A11 is a carcinoma-related protein located in the HCC exosomes [42], we speculated that expression of S100A11 might participate in EIF3C-mediated exosomes to promote HCC angiogenesis and tumorigenesis. Indeed, our results demonstrated that EIF3C upregulated expression of S100A11 in HCC cells PLC5 and SNU449 by Western blotting analysis (Figure 4A). After demonstration of knockdown efficiency of three S100A11 shRNAs by Western blotting analysis (Figure 4B), we found exosomes collected from EIF3C-expressed HCC cells significantly reduced HUVEC tube formation of *in vitro* angiogenesis assays (Figure 4C). Importantly, simultaneous high RNA expression of EIF3C and S100A11 in human HCC tumors showed worst survival, and expression of either EIF3C or S100A11 showed poor survival in compared to low expression of both RNAs of HCC patients in TCGA HCC dataset (Figure 4D). We also validated concordant protein expression of EIF3C and S100A11 on HCC tumor tissues by IHC assays. Consistently, the concordant high expression of EIF3C and S100A11 in HCC tumors showed the poor survival in compared to longer survival of low expression of both proteins in human HCC patients (Figures 4E and 4F).

## DISCUSSION

To extend our understanding how aberrant translational machinery participated in tumorigenesis, we investigated the roles of upregulated-EIF3C in association with poor patient survival and served as theranostic target for improving HCC therapy. We found that EIF3C mediated tumor progression via increasing release of oncogenic exosomes to potentiate angiogenesis and tumorigenesis in tumor microenvironment. Treatment of exosome inhibitor GW4869 or suppression of S100A11 expression to diminish EIF3C-mediated HCC angiogenesis further suggested that up-regulated EIF3C expression in HCC is a theranostic target for HCC therapeutic interventions.

Except EIF3E and EIF3F found down-regulated and conferred tumor suppressive activity in cancers [43, 44], majority of EIF3 members including EIF3A [45], EIF3B [46], EIF3C [47], EIF3D [48], EIF3H [49], EIF3I [50, 51] and EIF3M [52] were up-regulated and played important roles in tumor progression of multiple cancer types. Nevertheless, detail mechanisms of how the aberrant expression of individual EIF3s directly involved in the tumorigenic signaling pathways rather than simply accompanied with aberrant protein synthesis remain critical issues for elucidation. For examples, EIF3E also called *int-6* because of frequent integration by the mouse mammary tumor virus resulted in a truncated EIF3E protein that was shown to be oncogenic to induce mammary tumorigenesis [53, 54]. However, ectopic expression of full-length EIF3E did not cause cellular transformation in NIH3T3 cell [22]. EIF3F expression is frequently lower in 70∼100% of tumors in compared to the tumor tissues possibly due to frequent detection of loss of heterozygosity in cancer genome or interacting with and phosphorylated by cyclin-dependent kinase 11 (CDK11) during apoptosis resulted in suppression of protein translation during apoptosis [55, 56]. EIF3I can specifically upregulated and interact with phospho-Akt1 (Ser473) to prevent de-phosphorylation by phosphatase PP2A and to sustain oncogenic p-Akt1 activity to facilitate tumor progression [57].

Although we detected no increase of cell proliferation and decrease of cell migration in EIF3C-expressing HCC cells that are inconsistent to previous reports [17–19, 21, 23, 58], we revealed an EIF3C oncogenic mechanism via increasing release of exosomes in HCC cells to target surrounding HCC and stroma cells to promote HCC angiogenesis and tumorigenesis. With cross-validations of aberrant expression of EIF3C with endothelial markers for angiogenesis and simultaneous high expression of EIF3C and S100A11 in association with poor HCC patient survival in human HCC tissues, some interesting caveats are warrant to explore for better understanding the roles of EIF3C in HCC tumor angiogenesis and progression.

First of all, with lines of evidence of supporting EIF3C-stimulated release of exosomes determined by vesicle size and markers, we suspected that the increasing EIF3C exosomes are due to alteration of protein and RNA contents to promote HCC angiogenesis and tumorigenesis. Studies of underline mechanisms of EIF3C-stimulating exosome biosynthesis might lead to future clinical applications. Secondly, although we focused mainly on assays of EIF3C-exosomes mediated angiogenesis, how these EIF3C-exosomes increased EIF3C expression in Huh7 subcutaneous tumors in nude mice to mimic the observation of EIF3C upregulation in human HCC tissues and how EIF3C exosome mediated angiogenesis without affecting expression of HIF1A and VEGF in HCC cells require additional mechanistic studies. Finally, we demonstrated that S100A11 participated in EIF3C-exosomes mediated HCC angiogenesis and simultaneous high expression of EIF3C and S100A11 is associated with poor HCC patient survival. Omics approaches to reveal other tumorigenic components such as proteins, RNAs and non-coding RNAs in EIF3C-exosomes could further dissect the EIF3C upregulation mediated tumor progression.

Together, an oncogenic mechanism of enhancing exosome release by ectopic expression of EIF3C in HCC cells to promote HCC angiogenesis and tumorigenesis was revealed in this study. Although the detail molecular mechanisms for individual expression of EIF3C in tumor progression of HCC and other cancer types remain obscure, upregulated-EIF3C served as theranostic marker through treatment of exosome generation inhibitor GW4869 and suppression of S100A11 expression is firmly established. Future studies on using EIF3C as biomarker for anti-angiogenesis therapy in HCC and the combination therapy of anti-EIF3C exosomes with anti-angiogenesis therapies might be important for improvement of HCC intervention.

## EXPERIMENTAL PROCEDURES

### Cell culture

Hepatocellular carcinoma cell lines: PLC/PRF/5(PLC5) and SNU-449 were purchased from ATCC; and Huh7 was obtained from Japanese Collection of Research Bioresources (JCRB) Cell Bank. Cells were all cultured in DMEM (Gibco 12800-058) containing 10% fetal bovine serum, 100 units/mL penicillin, 100 μg/mL streptomycin, and Non-Essential Amino Acids Solution (Gibco 11140-050). Human Umbilical Vein Endothelial Cells (HUVEC) were purchased from ScienCell and cultured in Endothelial Cell Medium (ScienCell).

### Chemicals and reagents

PKH26 membrane dye and exososme inhibitor GW4869 were purchased from Sigma-Aldrich. Trans-well chambers were purchased from Merck Millipore. PrestoBlue Cell Viability Reagent were purchased from Invitrogen. jetPEI^®^ transfection reagent were purchased from Polyplus-transfection^®^. EIF3C polyclonal antibodies were purchased from Sigma. S100A11,CD63,TSG101,CD31,CD34,CD81,GM130,HA, Actin polyclonal antibodies were purchased from GeneTex; CD9 and ALIX polyclonal antibodies were purchased from Abcam, and horseradish peroxidase-conjugated goat anti-rabbit and anti-mouse secondary antibody were ordered from System Biosciences. pcDNA5/FRT EIF3C plasmid was kindly given by Dr. Hershey JW.

### Exosome isolation and purification

PLC5, SNU-449 and Huh7 cells were cultured to 70% confluence in 10 cm dishes in DMEM medium supplemented with 10% FBS, and transfection for 16 hr, then culture cells in serum free medium for 2 hr to collect their supernatants. Supernatants followed by filtration through a 0.2 μm filter from Millipore. The filtered supernatants were then concentrated by centrifugation using an Amicon® Ultra (AU) filter (Millipore). The final concentrated crude exosomes is ready for downstream analysis except western blot. We cultured cells (2 × 10^7^) in three 10 cm dishes and concentrated supernatants to 700 ul to perform angiogenesis, TEM, uptake and plug assays in each sample in every test. These crude exosomes were used within one week. The filtered supernatants were then mixed with Total Exosome Isolation Reagent (Thermo) and incubated overnight at 2° C to 8° C. The precipitated exosomes were recovered by standard centrifugation at 10,000 × g for 60 min. The pellet was then ready for protein extraction and performed western blot.

### MASS spectrometry

Proteins from Immuno-precipitation or exosomes were fractionated by one-dimensional electrophoresis followed by trypsin in-gel protein digestion and measure the peptide mass and peptide fragment mass by LC-ESI/MS/MS or 2D-LC-ESI/MS/MS and to identify the proteins with matching to database.

### Nanoparticle tracking analysis (NTA)

The number and size of nanoparticles was assessed using NanoSight NS300 nanoparticle characterization system by DKSH in Taiwan.

### Mice

Male NOD.CB17-*Prkdcscid*/NcrCrlBltw mice were purchased from BioLASCO Taiwan Co., Ltd and used at 4–6 weeks of age. Animal experiments abided by the guidelines for animal care and use issued by Academeia Sinica SPF animal facility

### Transmission electron microscopy (TEM)

Purified exosomes were fixed in 4% paraformaldehyde (w/v) in 200 mM phosphate buffer (pH 7.4). Fixed exosomes were dripped onto Formvar carbon-coated 200 mesh copper grids and absorbed at room temperature (RT) for 10 min. Excess liquid was removed with filter paper. Adsorbed exosomes were negatively stained with 3% phosphotungstic acid at RT for 5 min, dried with an incandescent lamp for 2 min, and observed via TEM (FEI Teccnai G2 F20 TWIN). Images were obtained using a cooled slow CCD camera.

### Western blotting

PLC5-, SNU-449- and Huh7-derived exosomes and cells were lysed in RIPA buffer containing protease inhibitors and proteins were resolved using SDS-PAGE. After transfer to a PVDF membrane, standard immunoblot analysis was performed. Membranes were incubated with primary antibodies HA (1:1000), Actin (1:1000), TSG101 (1:1000), CD63 (1:1000), EIF3C (1:1000), and S100A11(1:1000) at 4°C overnight in a buffer containing 5% skim milk, and then with a horseradish peroxidase (HRP)-conjugated secondary antibody at 37°C for 30 mins. Protein-band densities were analyzed quantitatively using Image Quant TL.

### Exosome fluorescent labeling and uptake assay

The final concentrated crude exosomes were labeled using the green lipophilic fluorescent dye, PKH26 for 5 min. PKH26-labeled exosomes were pelleted at 100,000 g for 70 min, washed three times with PBS, and re-suspended in Endothelial Cell Growth Medium. HUVEC cells were incubated with labeled exosomes for 16 h. Images were obtained using an inverted fluorescence microscope (PE Ultra VIEW).

### Cell proliferation

Cells were seeded on a new 96-well plate (5,000 cells/well) in DMEM medium with supplements. PrestoBlue^®^ was measured after 72 hr. Eight wells were used for each group at each representative test. Whole experiments were repeated three times.

### Cell migration

For the cell migration assays, cell culture was performed using 24-Well Millicell inserts (Millipore) according to the manufacturer’s instructions. Cells (1 × 10^5^) in 300 μl serum-free medium were added to the upper chambers and cultured for 24 h. Non-migrating or non-invading cells were removed with cottons swabs. Cells migrated or invaded to the bottom of the membrane were stained with the cell stain buffer and counted under microscope and photographed. Three independent experiments were performed for the same conditions.

### Tube formation assays in µ-slide angiogenesis

The slides were purchased from ibidi. Ten µl of Geltrex^®^ (Thermo) gel was applied to each inner well. For a final cell number of 10,000 cells per well, we adjusted a cell suspension of 2 × 10^5^ cells/ml. Then mixed exosome and ECM medium (1:1) thoroughly. Applied 50 µl cell suspension to each upper well. Immediately after seeding the cells, position the slide on an inverted microscope equipped with an incubation chamber and then start a time-lapse recording with 4× magnification and a time interval of 20 minutes in between the single images and data acquisition for 16 H (Model- Leica DMI 6000B).

### Plug assay

Mixed a total of 50 μl final concentrated crude exosomes with 250 μl ice-cold matrigel (the matrigel maintains as liquid form at 2–8° C and solidifies rapidly at 22–37° C). Subcutaneously injected the 300 μl matrigel mixture into a flank of five male NOD SCID mice (one injection site per mouse) with an ice-cold syringe and a 24 G one inch needle. After inoculation for 14 days, excised the matrigel, fixed with formalin overnight, embedded in paraffin, and sectioned onto slides.

### *In vivo* xenograft growth

5 × 10^6^ Huh7 cells in 1:1 ratio of Exosome: Matrigel (Growth Factor Reduced; BD Biosciences) were injected subcutaneously in flanks of 8-week-old male nude mice. Tumors were resected and weighed after 4 weeks. Formalin-fixed and paraffin-embedded tissue samples were examined with IHC stain.

### IHC and scoring

IHC was performed on formalin-fixed, paraffin-embedded tissue sections. De-paraffinization, antigen retrieval and antigen-antibody reactions were performed using an automated DAKO Envision with Dual Link system-HRP. Tissue sections were incubated with primary antibodies followed by HRP-conjugated secondary antibody (from DAKO envision kit). Primary antibodies EIF3C (1:100), CD31 (1:00), CD34 (1:100), and S100A11 (1:100) were purchased from Sigma and GeneTex. Staining was developed using counterstained with haematoxylin and evaluated by pathologist. The scores all from the pathologist’s visual estimation. We designed 4-grade EIF3C and S100A11 scoring criteria from 0 to 3. Score ranks usually lie in a range from “negative” to “positive”, which describing different force of IHC expression in investigated groups, include: “negative” (Score 0), “weak” (Score 1), “moderate” (Score 2), “strong” (Score 3). IHC marker for CD34, were used to establish micro-vessel density (MVD). This parameter is often presented as a number of micro-vessels per square millimeter or mean value with standard deviations. The MVD was measured based on Weidner’s method. Each positive endothelial cell cluster of immune-reactivity in contact with the selected field was counted as an individual vessel in addition to the morphologically identifiable vessels with a lumen. The intensity of the staining was scored as 0, 1, 2, 3, indicating absence of staining, weak, moderate, or strong intensity, respectively. Besides, we counted the absolute quantity of CD31 positively stained cells for each investigated in different experimental groups. Results in studies using this method to present as mean values of positively stained cells (and/or structures) among counted experimental groups.

### Real time PCR

For real time PCR, the total RNAs of PLC5, SNU-449 and Huh7 cells with overexpression of EIF3C or control were extracted by using TRIzol reagent (Invitrogen) and quantified by spectrophotometry (Nanodrop 2000; Thermo Scientific). The cDNA was synthesized using Revert Aid first strand cDNA synthesis kit (Thermo Scientific). The SYBR Green Supermix (Bio-Rad) was used for real time PCR on a 500 Fast & 7500 Real-Time PCR System (Thermo Scientific). Relative change in gene expression level was determined using the 2−ΔΔCt method. The primers were designed as follows: β-actin-Forward CTGGCACCCAGCACAATG, β-actin-Reversed CCGATCCACACGGAGTACTTG, VE GF-F GGGGGCAGAATCATCACGAA, VEGF-R GC AACGCGAGTCTGTGTTTT, TGF-β1-F CGTGGAG CTGTACCAGAAATA, TGF-β1-R TCCGGTGACATC AAAAGATAA, HIF1A-F CCAGTTACGTTCCTTCGATC AGT and HIF1A-R TTTGAGGACTTGCGCTTTCA.

### TCGA RNA

The RNA sequencing result of HCC and corresponding clinical data from The Cancer Genome Atlas (TCGA) was acquired from TCGA Data Portal in Nov. 2015. Tumor parts and adjacent normal parts are annotated as TCGA indication. The RSEM expression value are output from level 3 RNAseqV2 result for further analysis.

### Statistical analysis

Quantitative data are presented as means ± standard error of the mean (s.e.m.). Using GraphPad Prism 7 software to test for significant differences in qPCR, proliferation, tumor growth, and migration assays. Data were compared using Student’s *t*-test, Log-rank (Mantel-Cox) test, Bartlett’s test, or ANOVA test. *P <* 0.05 was considered significant.

## SUPPLEMENTARY MATERIALS FIGURES AND TABLES






