# Supplementary material for: EIF3C-enhanced exosome secretion promotes angiogenesis and tumorigenesis of human hepatocellular carcinoma
Source: Oncotarget. 2018 Jan 11;9(17):13193–205. doi: 10.18632/oncotarget.24149 (PMC5862571; doi:10.18632/oncotarget.24149)
Supplement: Supplementary file 1 [file oncotarget-09-13193-s001.pdf]

EIF3C-enhanced exosome secretion promotes angiogenesis and tumorigenesis of human hepatocellular carcinoma

SUPPLEMENTARY MATERIALS

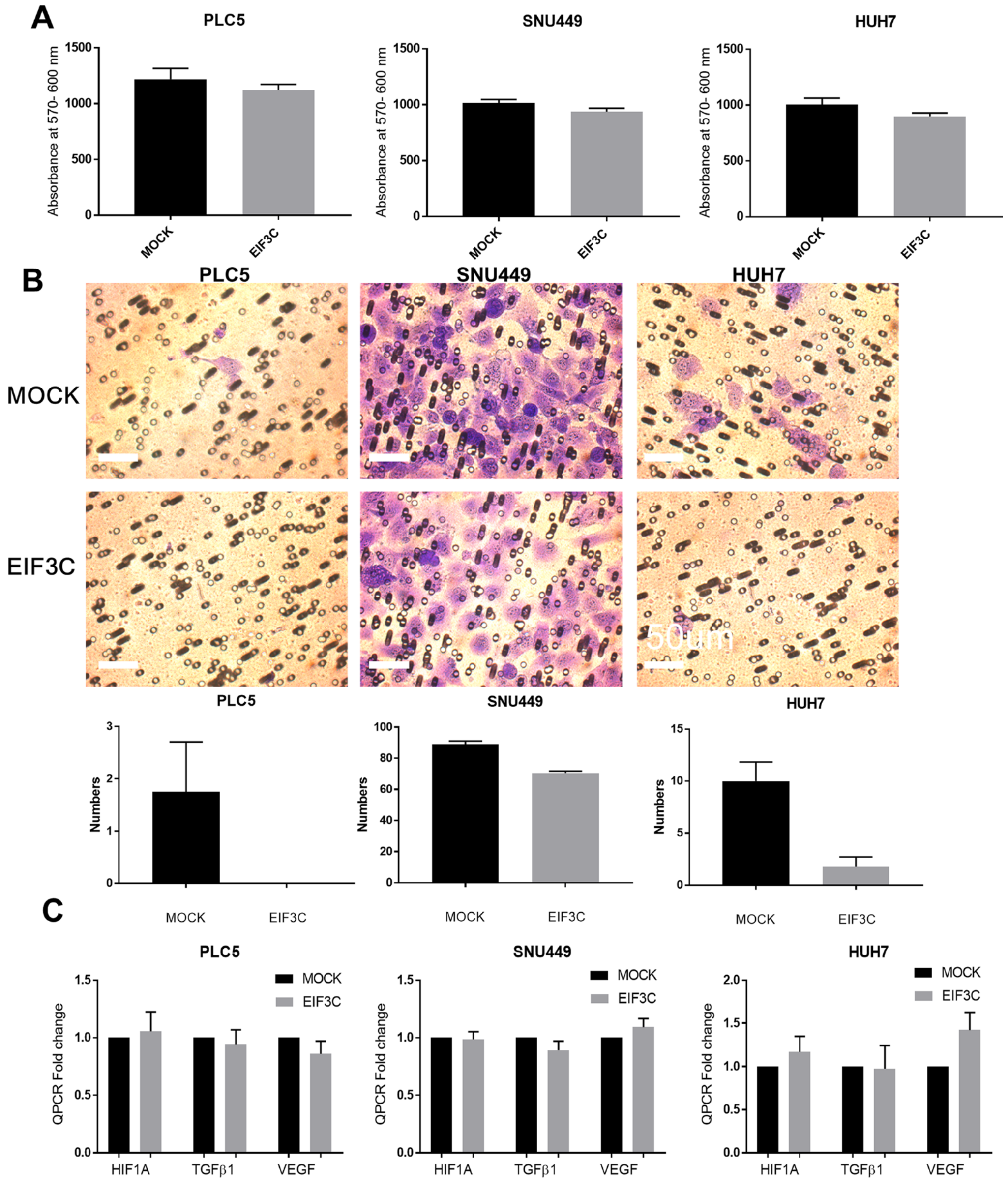

Supplementary Figure 1: Overexpressed-EIF3C did not alter cell proliferation, migration and expression of TGFβl and VEGF. (**A**) Expression of EIF3C in HCC cells PLC5, SNU449 and Huh7 did not alter cell proliferation rates in compared to that of parental cells. (**B**) Expression of EIF3C in HCC cells PLC5, SNU449 and Huh7 cells might slightly reduced trans-well cell migration . (**C**) Expression of EIF3C in HCC cells PLC5. SNU449 and Huh7 did not alter expression of migration-related genes such as HIFl A, TGFβ l and VEGF by quantitative RT-qPCR assays.

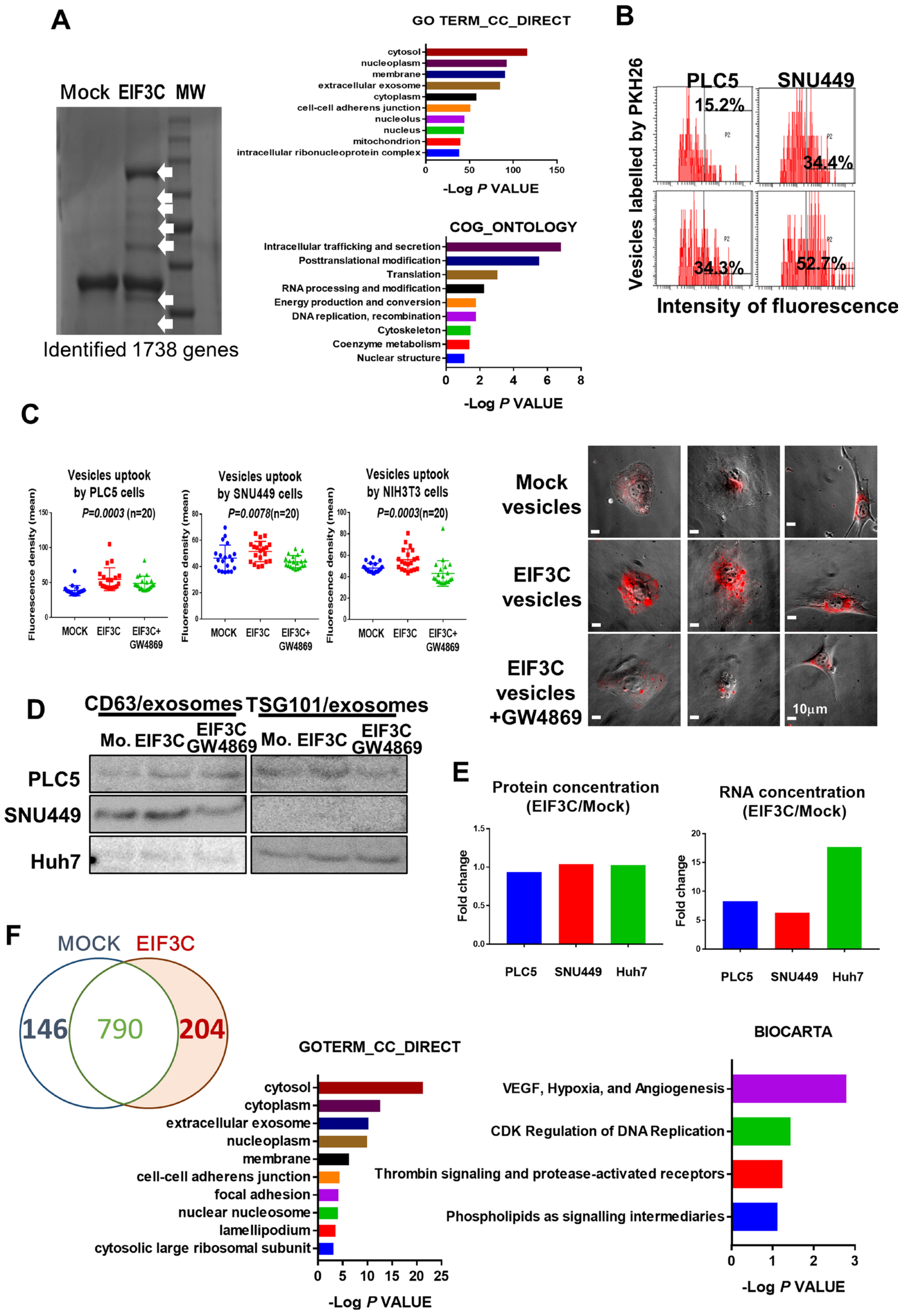

Supplementary Figure 2: Exploring molecular mechanisms of overexpressed-EIF3C and characterization of EIF3C-enhanced exosomes in HCC cells. (**A**) EIF3C immunoprecipitation-mass spectrometry in overexpressed EIF3C PLC5 cells (white arrows) gained 1738 gene and analyzed gene ontology and KEGG pathway of these genes in DAVID Bioinformatics Resources 6.8. (**B**) Uptook of PKH26 fluorescent labeled vesicles from EIF3C-expressed HCC cells in compared to that of mock vesicles of HCC cells. (**C**) HCC and NIH3T3 cells up took more PKH26 labelled vesicles from EIF3C-released than that of mock cells. (ANOVA summary). (**D**) Western blotting analysis of exosome markers CD63 and TSG 101 in purified exosomes of HCC cells with mock, EIF3C and EIF3C expression co-treated with GW4869 exosome generation inhibitor. (**E**) RNA and protein concertation ratios in exosomes of mock and EIF3C-overexpressed HCC cells. (**F**) Mass spectrometry analysis in compared between EIF3C and mock mediated PLC5 exosomes followed by gene ontology and BIOCARTA pathway analysis by DAVID Bioinformatics Resources 6.8.

Supplementary Table 1: List of 1738 EIF3C protein complex identified by immunoprecipitation and mass spectrometry.
See Supplementary_Table_1

Supplementary Table 2: List of exosome proteins (Entrez Gene ID) in PLC5 with and without expression of EIF3C.
See Supplementary_Table_2
